# Evidence generation for sustained impact in the response to gender-based violence: lessons from the SRHR Africa Trust Zimbabwe

**DOI:** 10.3389/fgwh.2023.1135393

**Published:** 2023-09-07

**Authors:** Raymond Mazhambe, Mildred Mushunje

**Affiliations:** Programmes Department, SRHR Africa Trust (SAT) Zimbabwe, Harare, Zimbabwe

**Keywords:** evidence, programming, GBV, reporting, police

## Abstract

Gender-based violence (GBV) is a pervasive problem in Zimbabwe, affecting women and girls. The types of GBV that are prevalent in Zimbabwe include sexual violence, intimate partner violence, and child marriage. The issue of evidence generation remains crucial in developing interventions that are tailor-made for GBV response. In an attempt to build pathways for the strengthening of GBV programming and to influence policy change, the Sexual and Reproductive Health and Rights (SRHR) Africa Trust (SAT) Zimbabwe conducted a secondary data review of GBV cases recorded in 2018 and 2019 in collaboration with the Zimbabwe Republic Police (Victim Friendly Unit) to understand the incidence of and the driving factors underlying GBV in Zimbabwe, including context-specific information with regard to sexual violence and an overview of perpetrator types. The study proffered recommendations that focus on the importance of improving GBV reporting and programming in Zimbabwe. The study establishes that the driving factors for physical violence include poverty, infidelity, and alcohol and drug abuse, while sexual violence is perpetrated by intimate partners and close relatives, among other driving factors.

## 1. Introduction

UN Women (1) estimates that one in three women will experience physical or sexual abuse in her lifetime. Gender-based violence (GBV), which is an umbrella term for harmful acts of abuse perpetrated against a person's will and rooted in a system of unequal power between women and men (2), undermines the health, dignity, security, and autonomy of its victims, yet it remains shrouded in a culture of silence. The specific forms of violence are defined as follows: sexual violence is any sexual act or attempt to obtain a sexual act by violence or coercion, or an act directed against a person's sexuality, regardless of the perpetrator’s relationship to the victim (Sexual Violence Research Initiative); and physical violence refers to the intentional use of physical force with the potential to cause death, injury, or harm (3). The Zimbabwe Demographic Health Survey (ZDHS) indicates that, in Zimbabwe, approximately one in three women aged 15–49 years has experienced physical violence and approximately one in four women has experienced sexual violence since the age of 15 (4). Sexual violence affects women and girls of all ages, with perpetrators often being partners or family members. Intimate partner violence (IPV) is a common form of GBV, with women often experiencing physical, emotional, and economic abuse from their partners (5). Child marriage is also prevalent, with one in three girls being married before the age of 18 (6). Despite the existence of legal frameworks (the Domestic Violence Act; the Sexual Offences Act under the Criminal Code) guided by the Constitution of Zimbabwe for the prosecution of GBV, such as Section 23 (which protects against discrimination based on sex, gender, and race), gender-based violence continues to affect many women, with most cases going unreported, and the fact that many of these women are suffering in silence undermines their basic fundamental human rights and freedom (7).

Zimbabwe's legal and policy framework on sexual and gender-based violence (SGBV), harmful practices (HPs), and sexual and reproductive health and rights (SRHR) includes the Constitution, the Domestic Violence Act (2006), the Criminal Law Codification and Reform Act, the Children's Act, the Labour Relations Amendment Act, the Trafficking in Persons Act, the National Gender Policy, the National Programme on GBV Prevention and Response, the National Action Plan on Ending Rape and Sexual Abuse, the Broad-Based Women Economic Empowerment Framework, and the Multi-Sectoral Protocol on the Management of Rape and Sexual Violence, among others. Section 52 (the Right to Personal Security) of the Constitution, for example, is particularly important to note because it guarantees every person the right to bodily and psychological integrity, which includes: (1) the right to freedom from all forms of violence from public or private sources; (2) the right, subject to any other provision of the Constitution, to make decisions concerning reproduction; and (3) the right not to be subjected to medical or scientific experiments, or to the extraction or use of bodily tissue, without their informed consent (7). There are still laws that need to be aligned to the Constitution.

Despite the growing awareness surrounding the issue of violence against women, it remains difficult to find reliable and harmonized data on the prevalence of gender-based violence (8). In their GBV gap analysis, the organization Enhancing Learning & Research for Humanitarian Assistance (ELRHA) (9) identifies the continued existence of a complex and diverse set of needs yet to be addressed relating to the protection of women and girls from gender-based violence. Gender-based violence continues to increase, especially against women and girls, and there are gaps in understanding of the factors contributing to this rise in GBV cases; hence, there is a need for context-specific interventions that will improve GBV programming and influence policy change. Therefore, the SRHR Africa Trust, in collaboration with the Zimbabwe Republic Police, conducted a secondary data review of gender-based violence cases between 2018 and 2019 in order to understand the driving factors of and the incidence of GBV in Zimbabwe, in a bid to proffer recommendations that will aid in the GBV response.

### 1.1. The socioeconomic situation in Zimbabwe

According to a report by the African Economic Outlook (10), Zimbabwe's economy was already in recession before the COVID-19 pandemic, contracting by 6.0% in 2019. According to the report, output fell because of economic instability and the removal of subsidies on maize meal, fuel, and electricity prices; suppressed foreign exchange earnings; and excessive money creation. It was also noted that the onset of the COVID-19 pandemic and continued drought led to a 10% contraction in real GDP in 2020. Inflation rose, averaging 622.8% in 2020, up from 226.9% in 2019. It was additionally reported that the foreign exchange reforms that were instituted in June 2020 dampened inflation, which had been raging at an annual rate of 838% in July. Fiscal and current account deficits also recovered after July, but both deteriorated for that year overall. The budget deficit increased from 2.7% in 2019 to 2.9% in 2020, while the current account moved from a surplus of 1.1% of GDP in 2019 to a deficit of 1.9% in 2020. The exchange rate depreciated by ZWL2.5 in February 2019 and stabilized at approximately ZWL82 to the US dollar in December 2020. The rate of poverty stood at 70.5% in 2019, while unemployment remained high, at a rate above 21% (10). The recession of the economy during this period has been accompanied by a rise in gender-based violence, with women and girls bearing the brunt of the burden. Against this backdrop, it can be noted that the segment of the population that suffers the most during times of crisis are young women, who need to fend for themselves and their children. Adolescent girls fall prey to child marriage, sexual exploitation, and commercial sex work. Hence, it is of paramount importance to put in place strategies for the socioeconomic empowerment of women in order to help alleviate poverty. Economically empowered women are self-reliant in supporting their families and rarely succumb to GBV arising from factors related to their livelihoods.

## 2. Study methods

This study employed a secondary data review approach to generate evidence. We define evidence generation as a multidimensional process that includes examination of the data source, study design, and degree of pragmatism; evidence generated in this way can be used to inform policies and programs and to build a body of knowledge (11). The approach made use of explicitly defined methods to identify, select, critically appraise, and extract and analyze data from the Zimbabwe Republic Police Victim Friendly Unit (VFU). The VFU is a department of the Zimbabwe Republic Police that was established specifically to police violence against women and children, particularly sexual offences and domestic violence.

Given the multiple GBV case files that were available for review, a secondary data review process was adopted to guide the study. This secondary data review process was conducted to review data sources obtained by the Victim Friendly Unit during the period of 2018 and 2019. The data sources reviewed covered all 10 provinces of Zimbabwe (Mashonaland Central, Mashonaland West, Mashonaland East, Harare, Bulawayo, Matabeleland North, Matabeleland South, Masvingo, Manicaland, and Midlands). The data collection process was carried out with support from 10 junior data clerks and the data were analyzed by research experts. The secondary data review process was conducted through a step-by-step process in the following manner. Step 1 consisted of framing of the questions for the review: an agreed set of questions to be answered by the review were crafted, drawing on the research objectives guiding the study. The study objectives were as follows:

• to establish the driving factors underlying GBV in Zimbabwe;
• to investigate prevalence data and trends over time in context-specific information with regard to GBV in Zimbabwe; and
• to assess types of perpetrator, stages of conflict, and the types of setting where GBV takes place.

The research questions were tailored to these guiding objectives, and included the general question: what are the driving factors of GBV in Zimbabwe? In addition, in relation to each specific case, the research questions included: who was abused; who was the perpetrator; where did the abuse occur; what were the circumstances surrounding the abuse; was the case reported to the police; when was the case reported (day/date) and at what time (on the day); and what is the current status of the case? These review questions guided the data review process. Step 2 consisted of identifying relevant work: after the questions were established, the data collection process began with review of the various GBV case files for Zimbabwe that were available from 2018 and 2019. This involved conducting an extensive inquest of the available data, focusing on the relevant questions guiding the study. Step 3 was to assess the quality of studies: during the data collection process, a rigorous review process was conducted to identify the cases available and their relevance to the research questions and to the requirements of the present study. This process was performed as part of an assessment of the quality of the research findings. A quality checklist guided by the research questions was developed. Step 4 consisted of summarizing and generating the evidence: the data extracted from the review process were synthesized into the form of descriptive statistics and thematic analysis was conducted; the results of this were organized into a systematic presentation of the data gathered in line with the main findings of the study. Finally, step 5 was to interpret the findings: the interpretation of the findings was organized around the thematic concerns arising from the study. A comprehensive analysis was conducted through assessment of the summarized data, and the emerging key thematic issues were identified in line with the research questions of the study.

## 3. Results

### 3.1. Relationships of victims and perpetrators

**Figure 1:**
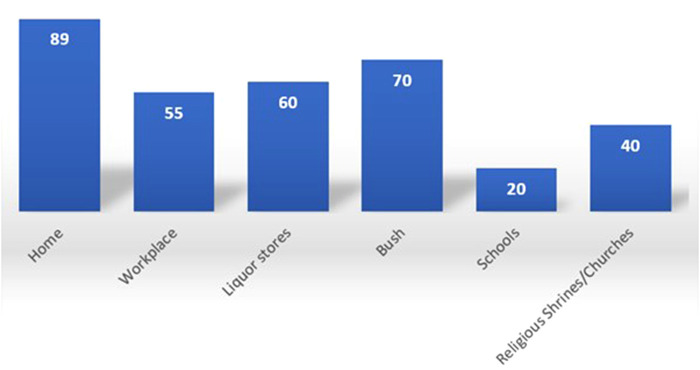
Settings in which GBV occurs (source: ZRP VFU: 2018–2019).

The results of the study undertaken by the SRHR Africa Trust (SAT) Zimbabwe, in collaboration with the Zimbabwe Republic Police Victim Friendly Unit, indicated that the relationships between victims and perpetrators varied, but included close family relationships, intimate partner relationships, and cases in which the victims and perpetrators were strangers. The findings revealed that, in most of the recorded cases of gender-based violence, the violence took place at home, in the workplace, in the bush, or at liquor stores, with a very limited number of cases occurring in schools and at churches or shrines (places of worship) (Figure 1).

**Figure 2:**
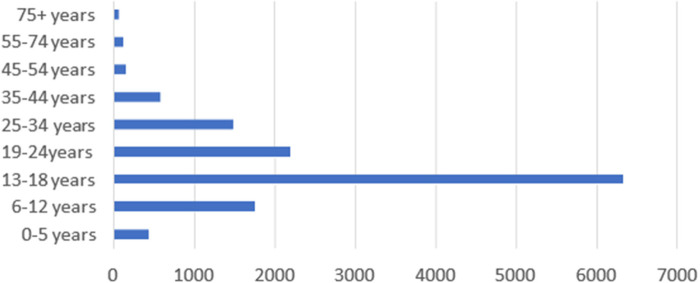
Age group distribution of the victims in reported cases of GBV (source: ZRP VFU: 2018–2019).

The victims were categorized into the following age groups: 0–5, 6–12, 13–18, 19–24, 25–34, 35–44, 45–54, 55–74, and 75+ years (Figure 2). Regarding sexual gender-based violence, a large number of cases in which the victim reported having experienced some form of sexual GBV were recorded in the 13–18 age category; in contrast, a large number of cases of physical violence were reported by victims in the 25–75+ age categories across the country.

### 3.2. Increase in cases of GBV against women and girls

**Figure 3:**
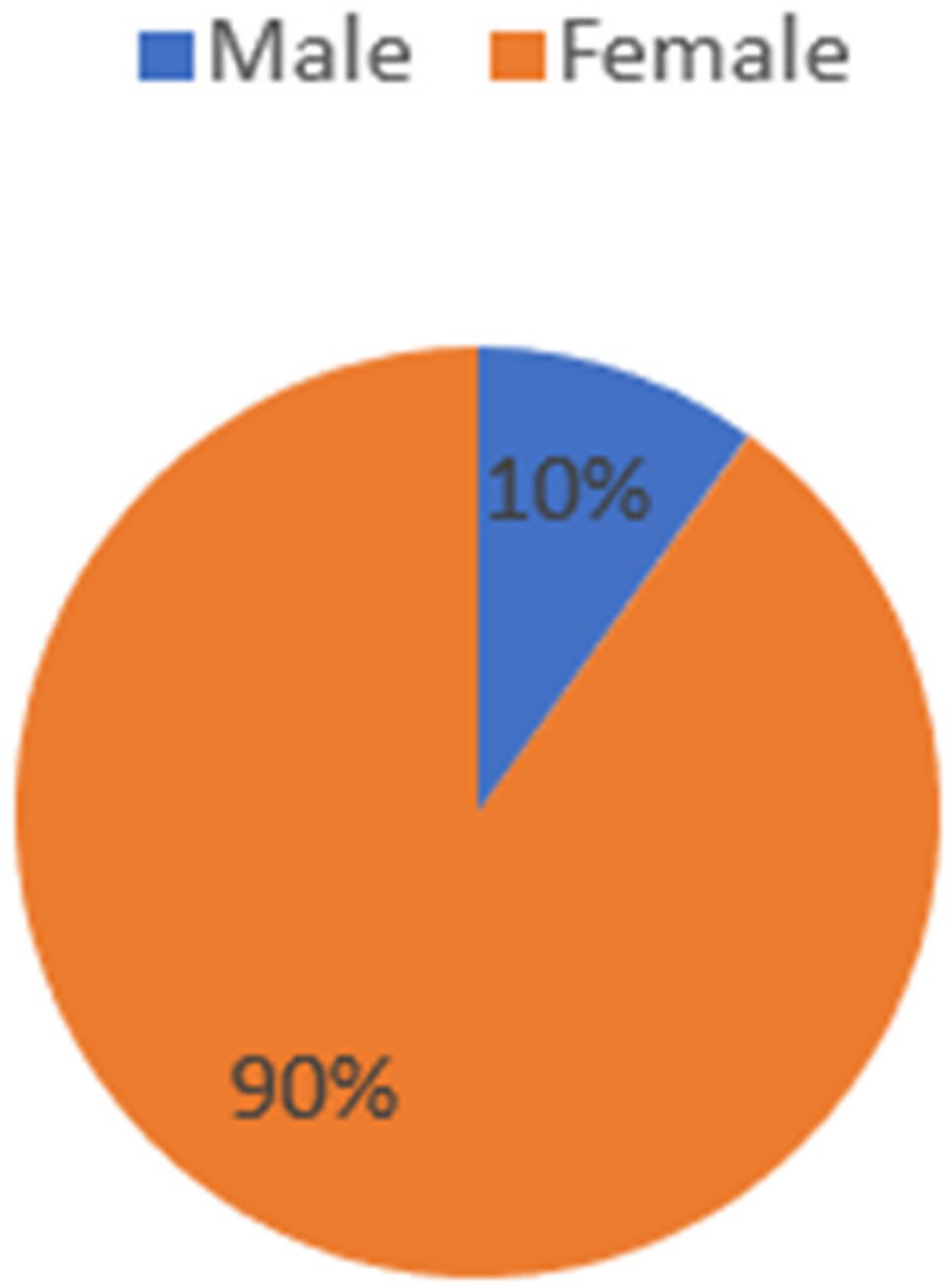
Gender distribution of the victims in reported cases of GBV (source: ZRP VFU: 2018–2019).

This study established that cases of GBV are increasing in Zimbabwe, and women and girls are the most affected. According to the findings, approximately 90% of all GBV cases reported on a daily basis are cases of women and girls reporting having experienced some form of violence, either physical or sexual gender-based violence (Figure 3). These findings are commensurate with a 2021 report by the United Nations Development Programme (UNDP), which established that the number of women and girls experiencing some form of sexual and physical violence is likely to increase as security, health, and money worries heighten tensions and strains and these are accentuated by cramped and confined living conditions (12).

### 3.3. Limited number of GBV cases reported by men

This study also found that there were a small number of recorded cases of men reporting having experienced some form of violence. It was discovered that, of every 100 cases, between three and five men reported having experienced some form of violence. In an analysis by Thobejane et al. (13), it was noted that men often do not speak out about their experiences of violence due to the stigma attached to being victims of such violence for them; therefore, this finding does not support the assumption that women are the only victims of GBV. Although men are also victims of GBV, available evidence suggests that the frequency, severity, and intensity of such violence are much greater for women (14).

### 3.4. Emerging issues

#### 3.4.1. Driving factors of physical violence and intimate partner violence

##### 3.4.1.1. Poverty/household economic challenges

The driving factors of GBV, especially physical violence, can largely be attributed to poverty. According to Arthur and Clark (15), economic dependence is a cause of domestic violence. A review of the circumstances contributing to cases of physical violence established that these involve economic challenges, such as lack of basic commodities in families, or marriages resulting in conflicts between family members or intimate partner violence. Brenner (16) also argues that economic insecurity has been found to be linked to the adoption of poor coping strategies, including substance abuse, and that these in turn have been found to be associated with various forms of gender-based violence. This research covered the pre-COVID era, but evidence arising from the COVID-19 era also substantiates the claim that economic household challenges contribute to GBV. UN Women reports that women's livelihoods were interrupted by lockdowns, given that many were informal traders (1). Under lockdowns, they could not generate income, and this resulted in the adoption of negative coping mechanisms, often resulting in violence.

##### 3.4.1.2. Infidelity

Infidelity also emerged as one of the factors contributing to an increase in physical violence, especially for women. The study indicated that a suspected case of infidelity, discovered by means such as going through a partner's phone, seeing WhatsApp messages from lovers, or finding a partner with a lover, resulted in physical violence across many communities in Zimbabwe. This finding resonates with numerous studies that have recorded an increasing number of cases of intimate partner violence, as highlighted by the United Nations Office on Drugs and Crime (UNODC), which has noted that almost 18% of women and girls aged 15–49 years who have ever been in a relationship have experienced physical or sexual violence from an intimate partner in the previous 12 months (17).

##### 3.4.1.3. Alcohol and drug abuse

Cases of alcohol and drug abuse were also a common factor contributing to a high number of instances of physical violence against women and girls. Richards (18) argues that economic strain, substance abuse, and isolation all tend to increase the risk of domestic violence, which comports with our study's findings. In recent years, Zimbabwe has witnessed a number of men and boys becoming involved in alcohol and drug abuse, who in turn become violent for various reasons, and women and girls become the victims in such circumstances.

##### 3.4.1.4. Misunderstandings among family members

Misunderstanding among family members was also another cause for concern contributing to physical violence. It was noted that most family members would fight over misunderstandings with regard to petty issues such as misplacement of tools or wrangling over property, among other issues. Reflecting on the foundations of these misunderstandings, Schneider et al. (19) argue that a violent outcome could be the result of male backlash resulting from feelings of emasculation and inadequacy over not being able to serve in the role of the breadwinner of the family. In the same manner, Bradbury-Jones and Isham (20) assert that such outcomes could also be the result of distorted power dynamics in the home, resulting in abuse and gender violence that escapes the scrutiny of anyone from outside.

#### 3.4.2. Circumstances of sexual violence

##### 3.4.2.1. Intimate partners and close relatives

The study affirmed that most gender-based violence, including cases of physical and sexual violence, is perpetrated by intimate partners, siblings, stepfathers against stepdaughters, or lovers (i.e., violence between a boyfriend and girlfriend). Krahe et al. (21) posit that girls and women continue to experience gender-based violence over the life cycle in homes, schools, churches, workplaces, the streets, and even therapeutic settings around the world. The findings indicated that some of the reported cases end up being withdrawn by the complainant because the perpetrator is the breadwinner of the family, and that close relatives often influence the decision to withdraw in these cases.

In cases of sexual violence, the study revealed that most male perpetrators take advantage of women and girls in their care. For example, stepfathers/fathers were found to be sexually abusing their daughters and uncles sexually abusing their nieces. In some cases, the findings indicated that women and girls seeking transport may be taken advantage of by perpetrators, as they end up taking them to the wrong destination and sexually abuse them along the way. When women and girls are on their way home from work or school, the findings revealed that they may also be sexually abused along the way. In other cases, the study established that lovers also commit date rapes, in which they take advantage of lovers whom they have gone out with to sexually abuse them.

Other factors include:

1. Walking distance from work/school
2. Seeking to use public transport
3. Date rape

Considering the reported cases, the study established that some of the women who reported their partners for gender-based violence withdrew their cases because of the fact that the perpetrators were often the breadwinners of the family. According to Alon et al. (22), increased economic dependence not only increases women's risk of gender-based violence but also makes it difficult for them to leave their perpetrators. The findings additionally revealed that pressure from relatives also resulted in the decision to withdraw in some cases. It was also established that the interplay of culture and religion also plays a part in influencing the decision to withdraw.

## 4. Discussion

The findings of the study indicate that evidence generation is of paramount importance in building pathways for a sustained GBV response. The term “pathways” refers to ways of achieving a specified result, or courses of action (23). The literature suggests that there is a paucity of information on GBV trends by context, which is critical in the establishment of interventions that will help prevent GBV in communities. The study findings show that understanding in-country GBV trends is important in identifying causal linkages relating to the occurrence of GBV. For example, poverty was identified as a triggering factor that is contributing to an increase in physical violence in Zimbabwe. As a result, there is a need to upgrade the country's safety nets for families and increase investment in household economic strengthening as a means of reducing physical violence in communities.

Issues of infidelity, misunderstandings, and alcohol and drug abuse, among others, were identified as the common factors contributing to the increase in gender-based violence in Zimbabwean communities. Against this backdrop, GBV programming needs to be strengthened in terms of its response to these key issues at the community level. “Programming” is defined as the designing, planning, and organizing of interventions to meet a particular need and improve a condition or situation (24). It is important to note that these emerging findings are significant in GBV programming, especially for civil society organizations and the relevant line ministries, such as the Ministry of Women Affairs, Community, Small and Medium Enterprises Development and the Ministry of Public Service, Labour and Social Welfare, in enabling the development of interventions and models that integrate families at the local levels and strategically engaging local leaders for a sustained response in the prevention of GBV.

The evidence also suggests that communities are not sufficiently empowered and well informed on the issues of GBV. Shockingly, the results also indicated that many men in society are ignorant of the laws against GBV. Although there was an increase in the recording of GBV cases reported between 2018 and 2019, women and girls remain trapped in harmful practices and religious doctrines that subjugate their fundamental freedoms and rights. The feminization of poverty and HIV in Zimbabwe, along with the high prevalence of SGBV and attitudes of acceptance toward it, are manifestations of deep-seated unequal power relations between genders and of the intersecting forms of discrimination that all women and girls experience, especially those in rural and marginalized communities. The number of cases that have been withdrawn by the complainants indicates that most of these women still lack self-agency and are not well informed about their constitutional rights when they have experienced any form of gender-based violence. It is imperative for civil society organizations, with support from line ministries, to engage and empower communities with knowledge and economic skills, which are critical for the protection of women and enable them to achieve upward social mobility even in the absence of their abusive partners.

The study findings also suggest that there is a need for a multi-sectoral approach to the GBV response. Maquibar et al. (25) highlighted the fact that the participants in their study considered GBV to be a serious social issue needing attention, and moreover needing a multipronged and multidisciplinary approach. It is important to note that communities need interventions that address the socioeconomic challenges that are contributing to the abuse and exploitation of women and girls. In this regard, sustained impact requires the implementation of an evidence-based program or policy that has lasting influence in a particular area or on a particular issue (26). It is important to note that these interventions require the active involvement of many actors. For example, engagement with religious and cultural leaders is critical, and they need to be at the forefront of movements to curtail the various forms of gender-based violence that are being perpetuated as a result of religious doctrines, patriarchal attitudes, and other harmful practices. It is important that service providers are also actively engaged and that they work collectively and establish one-stop centers where survivors of violence can obtain the support they need in one place. Engagement of civil society actors, parliamentarians, and line ministries enables them to work collectively in establishing pathways for improved legislation that can bring justice to communities and sets an example against any acts of gender-based violence. Therefore, it is critical that more studies be conducted and that evidence repositories are established to support a strengthened GBV response that will help in the development of interventions that are tailored to the needs of communities and in putting an end to GBV.

## 5. Conclusion

GBV remains a challenge in communities, and there is a need for robust measures and interventions that are curated from an evidence-based point of view. The lessons from the study conducted by SAT Zimbabwe in collaboration with the Zimbabwe Republic Police Victim Friendly Unit suggest that building evidence is a critical step in changing the narrative on GBV prevention efforts. The study has established the importance of understanding the driving factors of GBV, the settings in which it occurs, and the types of perpetrators involved, which are fundamental steps in the development of interventions against GBV in communities. It is important for more studies to be conducted to understand the factors contributing to GBV in various communities as steps toward reviewing policies and strengthening GBV programming.
